# Novel approach of encrypted network traffic classification using deep convolutional neural network with Artificial Bee Colony and Genetic Algorithm

**DOI:** 10.3389/fdata.2026.1821270

**Published:** 2026-04-28

**Authors:** Sujan Kumar Mohanty, Satyajit Rath, Satya Ranjan Sahu, Bikram Kumar Parida, Rakesh Chandra Balabantaray

**Affiliations:** 1 Academy of Scientific and Innovative Research, Ghaziabad, India; 2 Institute of Minerals and Materials Technology, Bhubaneswar, India; 3 Department of CSE, International Institute of Information Technology, Bhubaneswar, India

**Keywords:** Artificial Bee Colony, DCNN, encrypted traffic, feature selection, Genetic Algorithm, network traffic classification, optimization, VPN detection

## Abstract

The encode network traffic makes it difficult to perform successful and dynamic classification. This paper will present the use of a hybrid model to be used with the publicly available QUIC dataset to classify VPN and non-VPN encrypted traffic based on a Deep Convolutional Neural Network (DCNN) and Long Short-Term Memory (LSTM) network, which is optimized by the Artificial Bee Colony (ABC) and Genetic Algorithm (GA). The method involves multi-angle processing - preprocessing, Min-Max normalization, and features selection of with correlation analysis, Fisher Score, and mutual information to obtain a tiny, but meaningful feature set (Size, Batch Cache, Delta Previous Packet). The chosen features are translated to 2D tensors through a sliding time window of consecutive packets, which allows the spatio-temporal DCNN+LSTM architecture to represent the level of intra- and inter-packet feature associations as well as inter- and intra-packet time dynamics. The disadvantages of single-optimization are overcome using a dual metaheuristic optimization strategy again whereby the work of the global hyperparameter exploration is done using ABC and the structural optimization is done using GA. The imbalance of classes is reduced with weighted loss functions and stratified data division. The accuracy of the model is 99.66% with 0.994 ROC-AUC and 0.987 PR-AUC and its MCC is 0.963 which is even greater than that of the traditional classifiers (Decision Tree, Random Forest, SVM, KNN), individual deep-learning models (CNN, LSTM), and image-based FlowPic method. Three-quarters of stratified cross-validation marks the case of consistent generalization (99.53% ± 0.09% mean accuracy), and an ablation study confirms the value of any one of the components. The findings prove that the presented framework can be applied to monitor the network traffic on encrypted networks which are security-sensitive and in real-time.

## 1 Introduction

Traditional internet networks are being replaced by advanced management frameworks like Software-Defined Networking (SDN), driven by growing communication demands and enhanced hardware capabilities (Feamster et al., 2014). These developments have significantly increased computational demands on the network, making accurate network traffic classification essential for efficient traffic management and ensuring Quality of Service (QoS) (Anand and de Veciana, 2016).

Over recent decades, various approaches have aimed to predict quality, latency, and bandwidth requirements of network flows. A small set of flow features has proven effective for predicting key flow properties, whether through port numbers or statistical metrics (Nguyen and Armitage, 2008). Recently, deep learning (DL) models have gained traction for traffic classification, demonstrating high accuracy (Behrendt et al., 2017). However, most studies treat classification tasks as isolated, focusing narrowly on individual traffic types.

Our work is motivated by the evolving nature of network traffic and the realization that shared characteristics exist across different traffic classification tasks. This suggests that a unified model could adapt to diverse and dynamic traffic scenarios by leveraging common feature mappings (Jaiswal and Lokhande, 2013; Pereira et al., 2014; Zhang et al., 2015).

Although deep neural networks like DCNNs are computationally intensive, our proposed framework addresses these limitations. It enables multitask classification within centralized data centers with reduced computation time and memory usage. Moreover, our hierarchical architecture supports low-powered devices by allowing shared learning layers to be offloaded to the cloud or dedicated hardware. Local tasks then require only lightweight computation to complete specific classification objectives.

Though other works have used deep learning models that are optimized using the metaheuristic technique to classify network traffic, there are still significant drawbacks to the current methods. First, the current classification of encrypted traffic is based in most cases on the single-stage optimization, which tends to end up with the suboptimum solutions, as well as cannot effectively explore and exploit the search options. Second, single optimization techniques like standalone Genetic Algorithm (GA) or standalone Artificial Bee Colony (ABC) are likely to either over-explore (GA), or over-exploit (ABC), the search space but seldom do both. In the present work, ABC will be utilized to provide effective global search of the hyperparameter space (learnings rate, dropout ratio, LSTM units) and fine-tuning, and achieve impressive structural optimization, with the help of crossover and mutation, will be performed through GA. In this kind of dual-optimization strategy, there is extensive coverage of the search space that not only algorithm can cover. Third, compared to the current hybrid optimization methods, which usually are either based solely on feature selection (e.g., sequential feature selection) or feature hyperparameter optimization (e.g., directional feature integration), the proposed DCNN + ABC + GA framework conceives hyperparameter optimization (provided by ABC) and architectural optimization (provided by GA), thus, making the entire model tuning process more comprehensive and effective.

### 1.1 Key contributions of this work

- A hybrid DCNN-based framework, optimized with Artificial Bee Colony (ABC) and Genetic Algorithm (GA), is proposed for multitask traffic classification by extracting shared traffic knowledge.
- The proposed method demonstrates strong generalization capability across diverse classification tasks by leveraging prior training knowledge, yielding high accuracy and efficiency even on limited new data.
- A dual metaheuristic optimization strategy is introduced where ABC handles global hyperparameter exploration and GA performs structural optimization, addressing the limitations of single-optimization approaches in encrypted traffic classification.
- Comprehensive evaluation is performed using multiple metrics including ROC-AUC, Matthews Correlation Coefficient (MCC), and balanced accuracy to ensure reliability under class-imbalanced conditions.
- Ablation study is conducted to evaluate the individual contribution of each component (DCNN, LSTM, ABC, GA) to the overall model performance.

## 2 Related works

One of the most critical responsibilities in modern communication networks is the classification of network traffic. With the growing demand for high-throughput services and the increasing use of technologies like encryption and encapsulation, traditional classification methods have become less effective. As such, accurate classification is now essential for tasks such as anomaly detection, Quality-of-Service (QoS), traffic shaping, and network security.

Traffic classification has gained significant attention in both academic and industrial communities. Conventional methods typically relied on expert-defined rules and features. However, these are increasingly being replaced or supplemented by machine learning (ML) and deep learning (DL) techniques due to their ability to extract patterns from encrypted traffic and generalize across application types.

Early works targeted various practical problems—such as identifying elephant flows to mitigate congestion (Mori et al., 2004), and classifying P2P or multimedia traffic (Karagiannis et al., 2004), which often make up a large share of overall network usage. Researchers also emphasized that ignoring specific application-layer payloads can improve QoS depending on application requirements. Furthermore, early detection of malicious traffic significantly enhances network security (Anderson and McGrew, 2017).

In the supervised learning domain, algorithms such as k-Nearest Neighbor (kNN) (Kim et al., 2008), Naïve Bayes (Moore and Zuev, 2005), Bayesian Neural Networks (Auld et al., 2007), and Support Vector Machines (SVMs) (Este et al., 2011) were widely used. Decision Trees like C4.5 also found popularity (Li and Moore, 2007). In recent years, deep learning models have gained momentum (Lv et al., 2015), particularly due to their end-to-end learning capabilities, eliminating the need for manual feature engineering (Ma et al., 2019b).

However, not all models rely on supervised labels. For instance, (Bernaille et al. 2006) proposed an unsupervised learning approach, capable of handling unlabelled data, which is often the case in real-world scenarios.

Transfer learning enables adaptation between different but related domains, where training and testing data come from distinct distributions (Ma et al., 2019a; Pan and Yang, 2010). To address the domain gap, domain adaptation techniques are critical (Han et al., 2015a). Similarly, weakly supervised learning is becoming popular, where minimal labeling or proxy labels are used to improve performance across tasks.

Applications of such learning strategies span across fields. In computer vision, for example, unsupervised encoders have been used to isolate foreground from background (Han et al., 2015b), and multi-instance learning has been applied to co-saliency detection (Zhang et al., 2017). In natural language processing (NLP), multitask learning has been employed to boost tasks like named entity recognition and semantic annotation (Shin et al., 2016; Yang et al., 2017; Perera et al., 2018).

Recently, ensemble learning and domain transfer strategies have been explored to strengthen traffic classification (Sun et al., 2018; Moustafa et al., 2019). One-shot learning, where only a few labeled samples are used for training, was pioneered in (Fei-Fei et al. 2006) and is now gaining attention for traffic classification with limited ground truth.

A creative approach using packet size and inter-arrival intervals as features was introduced in (Roy et al. 2022), where Neural Ordinary Differential Equations (Neural ODEs) helped model temporal dynamics. Interestingly, (Lichy et al. 2023) shows that modern deep learning models may not always outperform classical machine learning models in malicious traffic detection.

Lastly, a powerful multitask model called Distiller was introduced in (Aceto et al. 2021), leveraging multimodal data and simultaneously learning across multiple tasks and traffic types. This approach outperforms earlier deep learning models by considering both intra- and inter-modal dependencies, thus better serving diverse provider needs in real-time traffic classification.

Recent developments have continued to take the encrypted traffic classification to further limits. To illustrate the advantage of using spatial and temporal feature extraction, (Lin et al. 2021) introduced a model, TSCRNN, a hybrid of CNN and RNN that is used to classify encrypted traffic that proves to be more efficient in this context. (Lotfollahi et al. 2020) described Deep Packet an end-to-end deep learning framework that encounters stacked autoencoders and CNNs in encrypted traffic characterization. Besides this, (He et al. 2022) also investigated the use of attention-based models in the classification of encrypted traffic and demonstrated that attention could enhance focus on features in more intricate traffic patterns. Transformer-methods also become available, with (Lim et al. 2019) suggesting a self-attention-based traffic classification model, which provides more effective dependencies among long-range variants than the sequential models traditionally. The current works show that the field of deep learning methods of encrypted traffic analysis has become more sophisticated, but the aspect of concomitant hyperparameter/architectural optimization has not been thoroughly discussed; this aspect justified the dual-optimization method, which the current work presents.

### 2.1 DCNN

**Figure 1:**
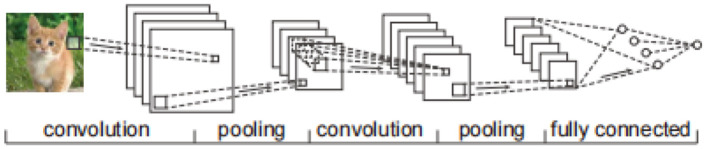
CNN architecture.

Convolutional Neural Networks (CNNs) are a class of deep neural networks particularly effective for data with grid-like structures, such as images (Lichy et al., 2023). A typical CNN architecture includes alternating layers of convolution, activation, pooling, and fully connected layers (see Figure 1). Each layer learns hierarchical representations, with higher layers capturing more abstract features.

#### 2.1.1 Convolution

**Figure 2:**
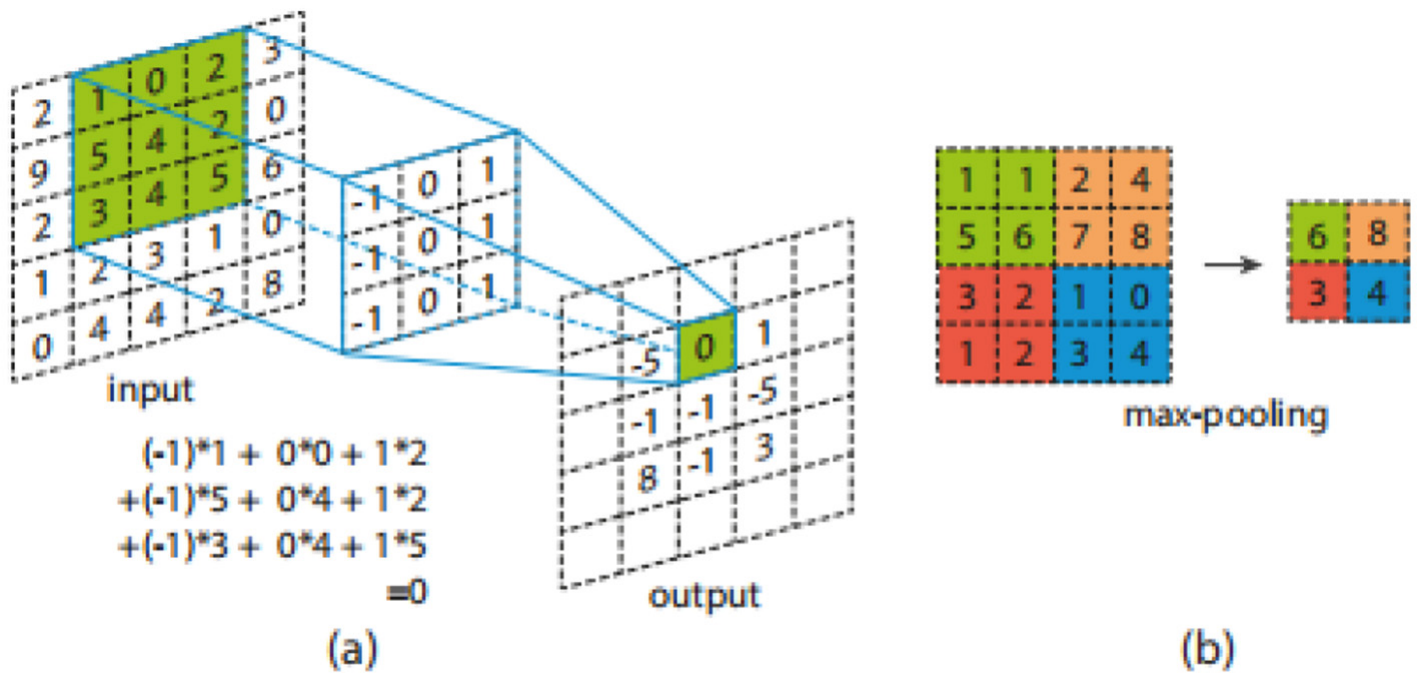
Representation of **(a)** convolution; **(b)** max-pooling.

Convolution applies a kernel (sliding window) across an image, generating output pixels as weighted sums of local regions. Shared weights enable learning of shift-invariant patterns. Figure 2a illustrates this operation.

#### 2.1.2 Activation functions

Non-linear functions are applied after convolutions to introduce non-linearity. The ReLU function, defined as *f* (*x*) = max(0, *x*), is widely used. For classification tasks, the softmax function is applied in the output layer to generate a normalized probability distribution.

#### 2.1.3 Pooling

Pooling reduces spatial dimensions, promoting translation invariance and reducing computation. Max pooling, shown in Figure 2b, selects the highest value from each local region.

#### 2.1.4 Normalization

Although not mandatory, Batch Normalization (Shin et al., 2016) is often used to speed up training and improve convergence by normalizing outputs across mini-batches.

#### 2.1.5 Loss functions

CNNs are trained to minimize a loss function, which measures the difference between predictions and true labels. Popular loss functions include Cross-Entropy Loss (with softmax), commonly used for classification, and Hinge Loss, often used in binary classification. Gradient descent, typically stochastic, is used to update weights during training (Pereira et al., 2014).

### 2.2 Artificial Bee Colony (ABC) optimization

The Artificial Bee Colony (ABC) algorithm, proposed by (Karaboga 2005), is a swarm-based optimization technique inspired by the foraging behavior of honey bees. It models three bee types: employed, onlooker, and scout. Solutions (food sources) are iteratively improved by local search and probabilistic selection. Poor solutions are abandoned and replaced with new random ones, allowing exploration and exploitation of the search space.

### 2.3 Genetic Algorithm (GA)

Genetic Algorithms (GA), developed by (Moore and Zuev 2005), mimic natural selection and evolution. They work with a population of candidate solutions that evolve through: (1) Initialization—Randomly generate individuals; (2) Evaluation—Assess fitness of each solution; (3) Selection—Choose the best individuals (e.g., via fitness-proportionate, rank-based, or Gaussian methods); (4) Crossover—Combine traits of selected individuals; (5) Mutation—Introduce random variations; (6) Replacement—Form the next generation based on fitness. The process continues until a termination criterion is met. GAs are robust and versatile, suitable for a wide range of optimization problems.

## 3 Methodology

This study proposes a hybrid deep learning model—DCNN + ABC + GA—to classify encrypted VPN and non-VPN traffic. The model pipeline includes data preprocessing, feature selection, anomaly detection, architectural design, parameter optimization, and performance evaluation.

### 3.1 Data preprocessing

The dataset of QUIC protocol records located in the publicly available QUIC dataset of the University of New Brunswick (UNB) was used, labeled, and included 13,260 QUIC protocol records. The dataset was recorded in a controlled laboratory setup where VPN and non-VPN traffic was made by using different applications (web browsing, video streaming, file transfer and VoIP) in connection to QUIC protocol. It was done by the creation of VPN traffic with the help of OpenVPN and WireGuard protocols to make sure that the VPN tunneling methods were variable. There are 2 basic classes of traffic: VPN (1,263 samples) and non-VPN (11,997 samples) traffic in the dataset whereby each traffic type comprises of different types of applications.

Even though the dataset of 13,260 records is relatively small by the standards of deep learning models, the given approach counterbalances that limitation with various techniques: (1) the feature set size is low (five features), which decreases the chances of overfitting the small sizes; (2) the data is augmented by anomaly detection and data preprocessing, which also improves the quality of the effective training data; (3) deep learning hybrid models of ABC-GA are efficient in searching the hyperparameter space, which does not involve with the large volumes of training data; and (4) drop.

In every record feature available are Timestamp, Delta Previous Packet, Size, Label, and Batch Cache. The steps of preprocessing involved unification of data type, missing values and Min-Max normalization. The labels (VPN:1, non-VPN:0) were digitized and schema validation made sure that they were ready to be trained.

### 3.2 Feature selection

Correlation analysis, Fisher Score and mutual information (MI) were exploited to select the features. According to the overall outcomes of the all three approaches, the ultimate feature set that is picked is Size, Batch Cache, and Delta Previous Packet. These characteristics became the most educative in terms of separating VPN and non-VPN traffic.

It is necessary to explain that though the Label column is present in the feature selection analysis (correlation, Fisher Score and MI plots), it is not taken as an input feature but just as a target variable (dependent variable) in the model training. It has been included in the analysis to determine the discriminative linking of each feature toward the classification target. Timestamp option has been eliminated since it was continually indicated by all three feature selection methods, that it had the lowest correlation with the target variable, lowest Fisher Score and the lowest mutual information score, which implies a low predictive value. Adding Timestamp as a second dimension would reduce classification performance without adding any additional information either and additional dimensionality would increase computation and overfitting.

### 3.3 Anomaly detection

To improve data quality, KMeans clustering was used on normalized Size, Batch Cache, and Label fields to detect and eliminate outliers. Cases far from cluster centers were flagged as anomalies, enabling cleaner training and increased model robustness.

### 3.4 DCNN architecture

The model integrates a DCNN with LSTM for spatial-temporal learning. It uses Conv2D and MaxPooling2D for spatial extraction, followed by BatchNorm, ReLU, and dropout for regularization. A 52-unit LSTM captures temporal dependencies, feeding into dense layers with sigmoid activation. The model comprises 101,473 trainable parameters and is configured to be trained using the Adam optimizer with binary cross-entropy loss function.

The detailed architectural and hyperparameter configuration is as follows: The Conv2D layer uses 124 filters with a kernel size of (1 × 3), stride of 1, and padding set to “same”. The MaxPooling2D layer uses a pool size of (1 × 2). The dropout rate is set to 0.3. The LSTM layer consists of 52 units with tanh activation. The final dense layers have 40 and 1 units respectively, with ReLU and sigmoid activations.

Even though the chosen set of the features is three (Size, Batch Cache, and Delta Previous Packet) as the final choice, the DCNN + LSTM usage is justified by the following purposes. The raw tabular features are first converted to a 2D representation in the form of a tensor representation that can be processed through convolutional processing. Namely, network packets are packed into sequences based on a sliding 10 consecutive packets the Timestamp and Delta Previous Packet values. Each sequence belongs to a 2D input array of size (10, 3), with 10 denoting the temporal window size (i.e., number of conjoining packets) and 3 denoting the dimensions of the features. This reshaping transforms the tabular data to a grid format where conv2d layer can help identify the local spatial patterns across features in each packet as well as in the wave of successive packets in the sequence. Second, brute force approaches like Random Forest or XGBoost might provide competent accuracy on single packet-based characteristics, however, the DCNN + LSTM design can reflect inter-packet relationships as well as time dynamics, which play a fundamental role in separating VPN tunneling patterns and normal traffic. The sequential nature of packets and their temporal relations with each other is particularly modeled using the LSTM layer and cannot be expressed using flow-level statistical features. Third, the convolutional layers provide automatic feature extractors, which learn higher-order feature interactions (e.g., the dependency between changes in packet size and inter-arrival time patterns) to be automatically learned, therefore complementing and not conflicting with the step of feature selection. The noise and dimensionality reduction is done by the feature selection, and the learner of the DCNN is the complicated non-linear combinations of the informative features that the feature selection has chosen.

### 3.5 Optimization with ABC and GA

The Artificial Bee Colony (ABC) algorithm is employed for hyperparameter tuning, while the Genetic Algorithm (GA) is used for structural optimization of the network architecture. ABC explores the hyperparameter search space through its foraging-inspired mechanism, and GA refines the architecture through crossover and mutation operations. Table 1 shows the ABC hyperparameter search space and optimal values found during optimization, and Table 2 presents the configuration parameters used for the Genetic Algorithm optimization.

**Table 1:** ABC hyperparameter search space.

Hyperparameter	Search range	Optimal value
Learning rate	0.0001–0.01	0.001
Dropout ratio	0.1–0.5	0.3
LSTM units	16–128	52
Batch size	16–128	32
Number of conv filters	32–256	124
Colony size	20	–
Max iterations	50	–
Limit (abandonment)	10	–

**Table 2:** GA configuration parameters.

Parameter	Value
Population size	30
Number of generations	50
Crossover rate	0.8
Mutation rate	0.1
Selection method	Tournament selection (*k* = 3)
Crossover type	Single-point
Elitism	Top 2 individuals preserved

### 3.6 Model training and evaluation

This model was trained in 50 epochs on Adam optimizer and binary cross-entropy loss. The effective ways to stop overfitting were early stopping (patience = 10 epochs) and the dropout regularization. The classification-appropriate measures such as accuracy, precision, recall, *F*1-Score, ROC-AUC, Precision-Recall Area Under Curve (PR-AUC), Matthews Correlation Coefficient (MCC), and balanced accuracy were used to measure model performance. Regression-based measures [Mean Absolute Error (MAE), Mean Squared Error (MSE)] were also not considered since they cannot be applied to binary classification exercises. Distribution of the detailed performance results is given and discussed in Section 4.

Since there was a large difference in the number of non-VPN and VPN samples (11,997 vs. 1,263), the weighted mean was used to train the model with a higher weight on the minority VPN population due to the resultant under-representation. In particular, the weight of the class of VPN was set to about 4.75 (majority of minority class) so that the model would measures the misclassification of the VPN traffic more severely. Also stratified splitting was carried out to preserve class proportions in both training and testing sets (80:20 split) as well as performance measures such as balanced accuracy, MCC and ROC-AUC to show performance reporting is imbalance aware.

### 3.7 Experimental setup

The experimental environment was made in such a way as to allow intensive training, reproducibility and effective performance of the model. The code was executed with Python 3.9 along with TensorFlow 2.10 and Keras 2.10 on a high-performance computing platform of an NVIDIA Tesla V100 GPU with 32 GB RAM and Intel Xeon E5-2690 v4 processor, regular system software being Ubuntu 20.04 LTS. The model has been trained with Adam optimizer with learning rate of 001, a batch size of 32 and a limit of 50 epochs, and early stopping through a patience of 10 epochs to avoid over-fitting. To preserve the class distribution an 80: 20 stratified train-test split was employed and the overall training time was around 45 min, including optimization. A full description of the configuration of the experiment is presented in Table 3 so that transparency and reproduction of the experimental set up would be possible.

**Table 3:** Experimental setup and configuration.

Parameter	Specification
Hardware	NVIDIA Tesla V100 GPU, 32 GB RAM
CPU	Intel Xeon E5-2690 v4 @ 2.60GHz
Operating system	Ubuntu 20.04 LTS
Software framework	TensorFlow 2.10, Keras 2.10
Programming language	Python 3.9
Batch size	32
Learning rate	0.001 (Adam optimizer)
Epochs	50 (with early stopping, patience = 10)
Train-test split	80:20 (stratified)
Training time	~45 min (including optimization)

### 3.8 Benchmark comparison

Four traditional classifiers were used for baseline comparison: Decision Tree provided interpretability and baseline classification; Random Forest evaluated ensemble learning benefits and feature randomness; SVM tested effectiveness in identifying non-linear patterns; KNN (*k* = 5) assessed instance-based classification using Euclidean distance.

In addition to traditional classifiers, recent deep learning-based encrypted traffic classifiers were also included for comparison: a standalone CNN-based classifier, an LSTM-based sequential classifier, and the FlowPic method (Shapira and Shavitt, 2019) which converts traffic into image representations. These additions provide a more comprehensive evaluation against state-of-the-art methods in encrypted traffic classification.

All models were trained on the same preprocessed feature set and evaluated using identical metrics to ensure a fair and consistent comparison. The comparative results are reported in Section 4.

## 4 Results and discussion

### 4.1 Data preprocessing and normalization

Initial preprocessing involved cleaning and structuring the raw QUIC files to retain five key features: Timestamp, Delta Previous Packet, Size, Label, and Batch Cache. Due to inconsistent data types and formats, especially in Delta Previous Packet and Batch Cache, normalization was required before model training.

As illustrated in Table 4, the raw dataset included a mix of numerical formats such as exponential timestamps and unscaled values.

**Table 4:** Sample of raw dataset.

Timestamp	Delta previous packet	Size	Label	Batch cache
1522953991	0	1,412	1	0
1522953991	0.000325	972	1	0.000325
1522953991	0.026619	1,412	0	0.026619
1522953991	0.027489	103	1	0.027489
1522953991	0.027745	97	1	0.027745

All numerical features were normalized to the [0, 1] range using Min-Max scaling, and the Label column was encoded (non-VPN as 0, VPN as 1) using LabelEncoder. The transformed dataset is shown in Table 5.

**Table 5:** Dataset after normalization and encoding.

Timestamp	Delta previous packet	Size	Label	Batch cache
1522953991	0	1,412	1	0
1522953991	0.000325	972	1	0.00023
1522953991	0.026619	1,412	0	0
1522953991	0.027489	103	1	0.015778
1522953991	0.027745	97	1	0.015852

Data type consistency was validated prior to training. The schema details, including column data types and completeness, are summarized in Table 6.

**Table 6:** Schema summary of pre-processed dataset.

Column	Non-null count	Data type
Timestamp	13,260	int64
Delta previous packet	13,260	int64
Size	13,260	int64
Label	13,260	object
Batch cache	13,260	float64

**Figure 3:**
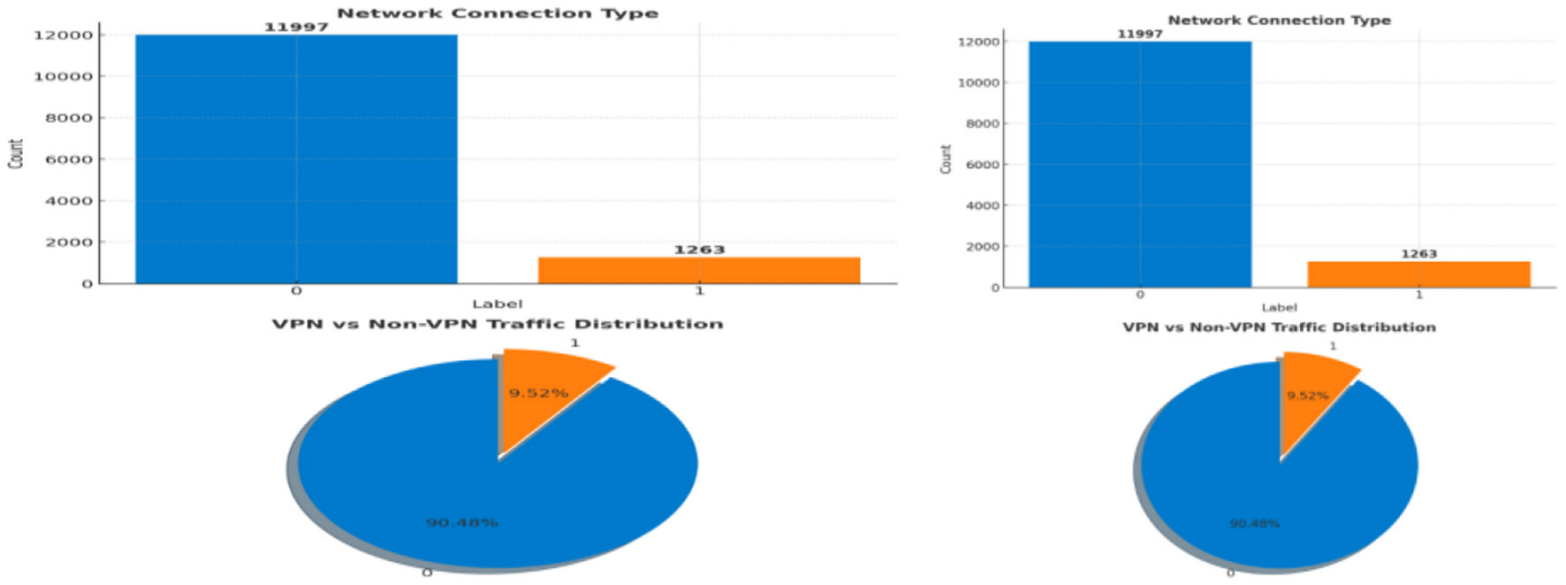
Normalization of labels using label encoder.

To evaluate class distribution, Figure 3 presents both bar and pie charts of the Label column. Out of 13,260 records, 11,997 samples were non-VPN and only 1,263 were VPN, indicating a significant class imbalance. This justified the adoption of performance metrics that account for bias toward the majority class.

Through normalization, encoding, and structure validation, the dataset was prepared for effective model training and performance optimization.

### 4.2 Feature selection and extraction

To improve model performance and reduce complexity, feature selection was performed using three statistical techniques: correlation analysis, Fisher Score ranking, and Mutual Information (MI).

**Figure 4:**
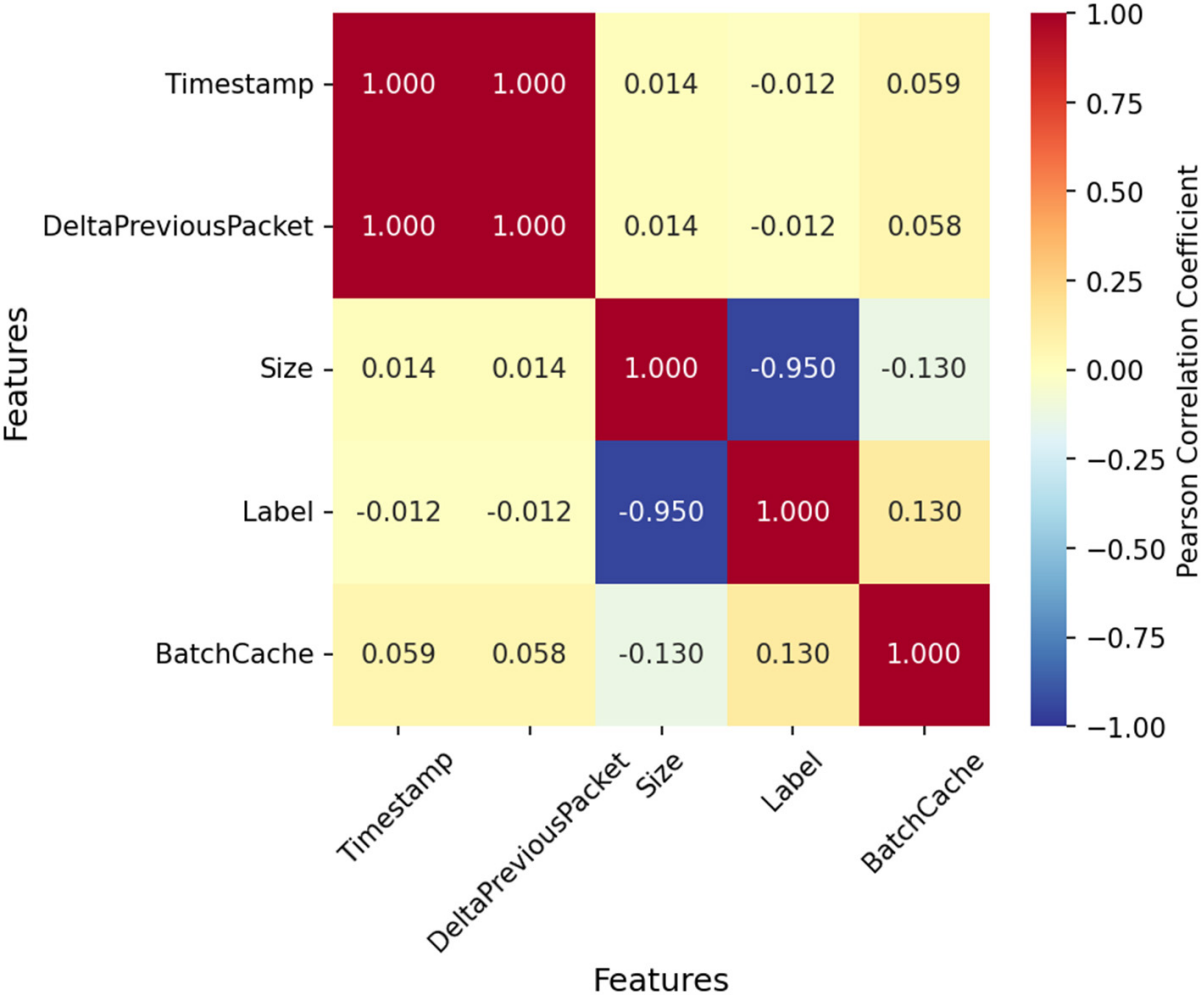
Correlation coefficient heat map.

Correlation analysis (Figure 4) revealed a strong negative correlation between Size and Label, indicating that packet size is a key feature for distinguishing VPN from non-VPN traffic. Conversely, Timestamp showed weak correlation with other variables, suggesting limited predictive value. Delta Previous Packet and Batch Cache had minimal interrelation, supporting their inclusion as distinct features.

**Figure 5:**
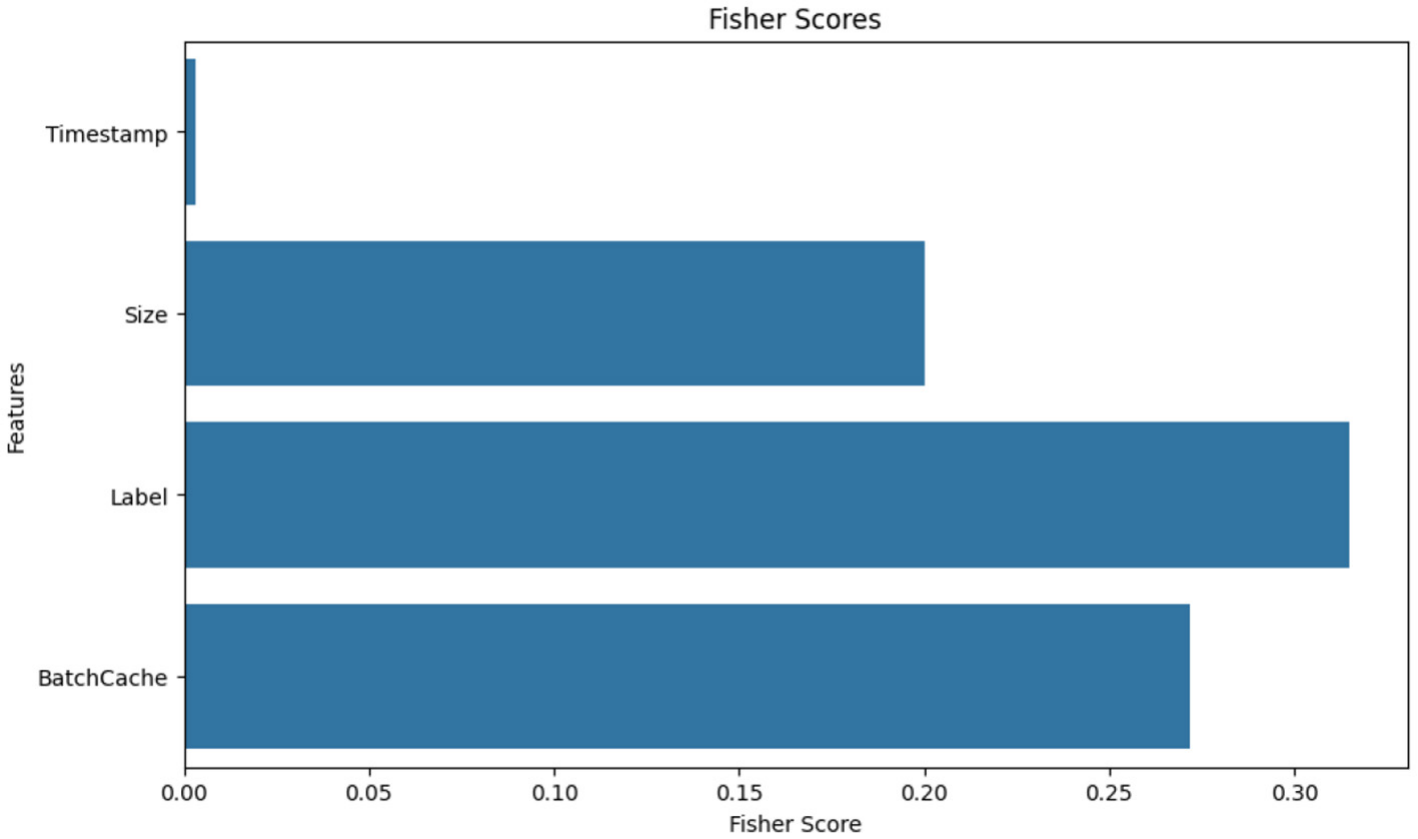
Fisher score ranking.

Fisher Score analysis (Figure 5) reinforced that Size, Label, and Batch Cache offer the most discriminative power for class separation. Again, Timestamp appeared redundant, contributing little to classification effectiveness.

**Figure 6:**
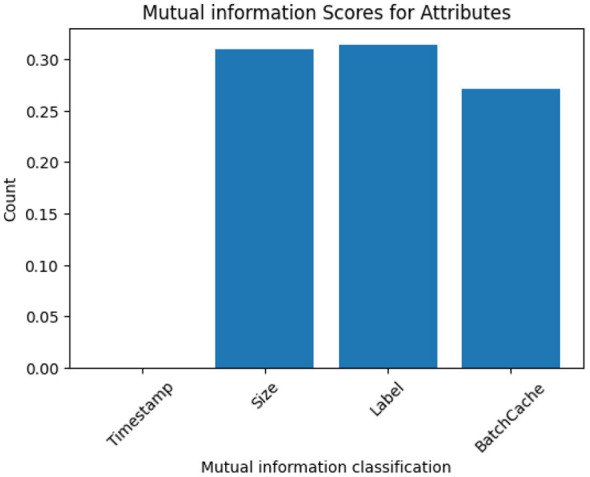
Mutual information classification plot.

Further, Mutual Information (MI) scores (Figure 6) confirmed the relevance of Size, Label, and Batch Cache, with Timestamp again ranking lowest in predictive contribution.

Based on these combined analyses, Size, Batch Cache, and Delta Previous Packet were selected as the most impactful input features, while Timestamp was excluded to streamline the model and reduce training overhead. This targeted selection helped maximize classification accuracy and minimize overfitting risk during deployment.

### 4.3 Anomaly detection

Following feature selection, an anomaly detection step was introduced to enhance data integrity and prevent misclassification caused by irregular or deceptive encrypted entries. Unsupervised KMeans clustering was employed using the normalized Size, Batch Cache, and Label features.

**Figure 7:**
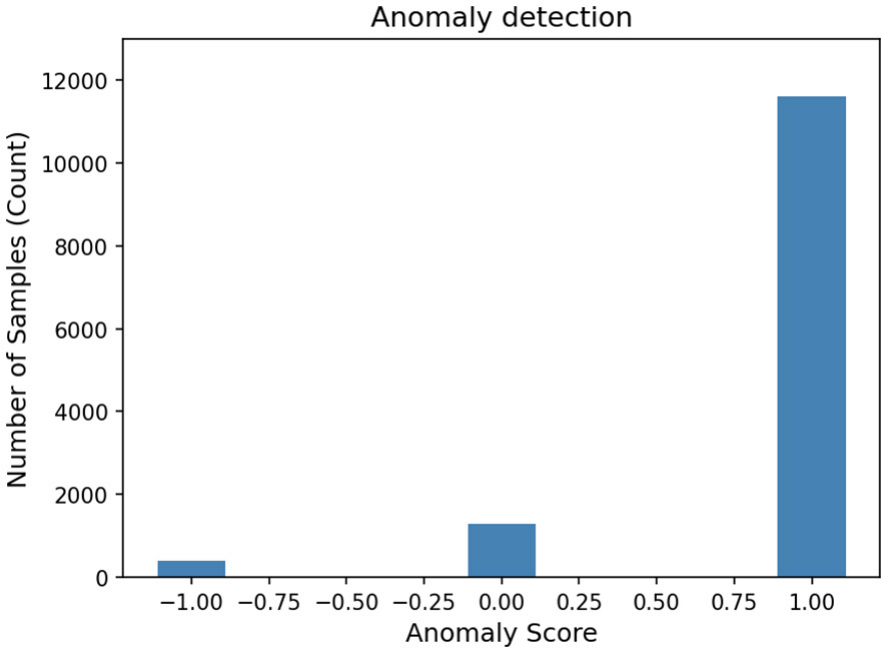
Anomalies detection using clustering approach through KMeans.

Each data point was scored based on its distance from cluster centroids to assess its normality. As illustrated in Figure 7, clustering distinguished between common and uncommon patterns, identifying significantly deviant points as anomalies.

The results of clustering assigned one of three labels: “1” for anomalous points, “0” for normal entries, and “1” for uncertain or borderline cases. Table 7 presents a sample of the dataset after anomaly labeling using KMeans clustering.

**Table 7:** Final dataset before training.

Timestamp	Size	Label	BatchCache	Anomalies
1522953991	1,412	1	0	1
1522953991	972	1	0.00023	−1
1522953991	1,412	0	0	1
1522953991	103	1	0.015778	0
1522953991	97	1	0.015852	0

This anomaly tagging provided two major advantages: (1) Problematic records could be excluded or handled separately, reducing noise in model training; and (2) Model robustness improved, ensuring consistent performance even when presented with distorted or irregular VPN traffic data. Incorporating anomaly detection significantly boosted dataset quality and made the subsequent classification stages more reliable.

### 4.4 Model development and configuration

Following preprocessing, feature selection, and anomaly detection, a customized deep learning model was developed to classify encrypted VPN and non-VPN traffic. The proposed architecture integrates Deep Convolutional Neural Networks (DCNN) with Long Short-Term Memory (LSTM) layers to capture both spatial and temporal characteristics of traffic data from the QUIC protocol dataset.

The model begins with a Conv2D layer to extract local patterns, followed by a MaxPooling2D layer to reduce dimensionality and mitigate overfitting. A BatchNormalization layer stabilizes training, and ReLU activation introduces non-linearity. A Dropout layer is used for regularization by randomly disabling a fraction of neurons during training. The LSTM layer with 52 units captures time-based dependencies between packets, which is critical for encrypted traffic. Finally, two Dense layers—including an output layer with sigmoid activation—classify each sample as VPN or non-VPN. Table 8 details the DCNN model architecture with layer configurations and parameter counts.

**Table 8:** DCNN model architecture.

Layer name	Output shape	Parameters
Conv2D	(1, 3, 124)	2,120
MaxPooling2D	(1, 3, 124)	0
BatchNormalization	−128	512
Activation (ReLU)	−128	0
Dropout	(3, 124)	0
LSTM	−52	36,816
Dense	−40	2,120
Dense (output)	−1	41

The model contains 101,473 trainable parameters, optimized using the Adam optimizer and trained with the binary cross-entropy loss function. The normalized feature set was used during training.

Initial results showed good convergence, validating the model structure and paving the way for metaheuristic optimization discussed in the next section. The hybrid use of convolutional and LSTM layers enabled the model to efficiently learn traffic patterns with modest computational resources, making it suitable for real-time encrypted traffic classification.

### 4.5 Optimization using ABC and GA

To further enhance the accuracy and performance of the DCNN model, a hybrid metaheuristic optimization approach was applied, combining the strengths of the Artificial Bee Colony (ABC) and the Genetic Algorithm (GA). This combination enabled efficient tuning of hyperparameters and structural optimization of the model.

**Figure 8:**
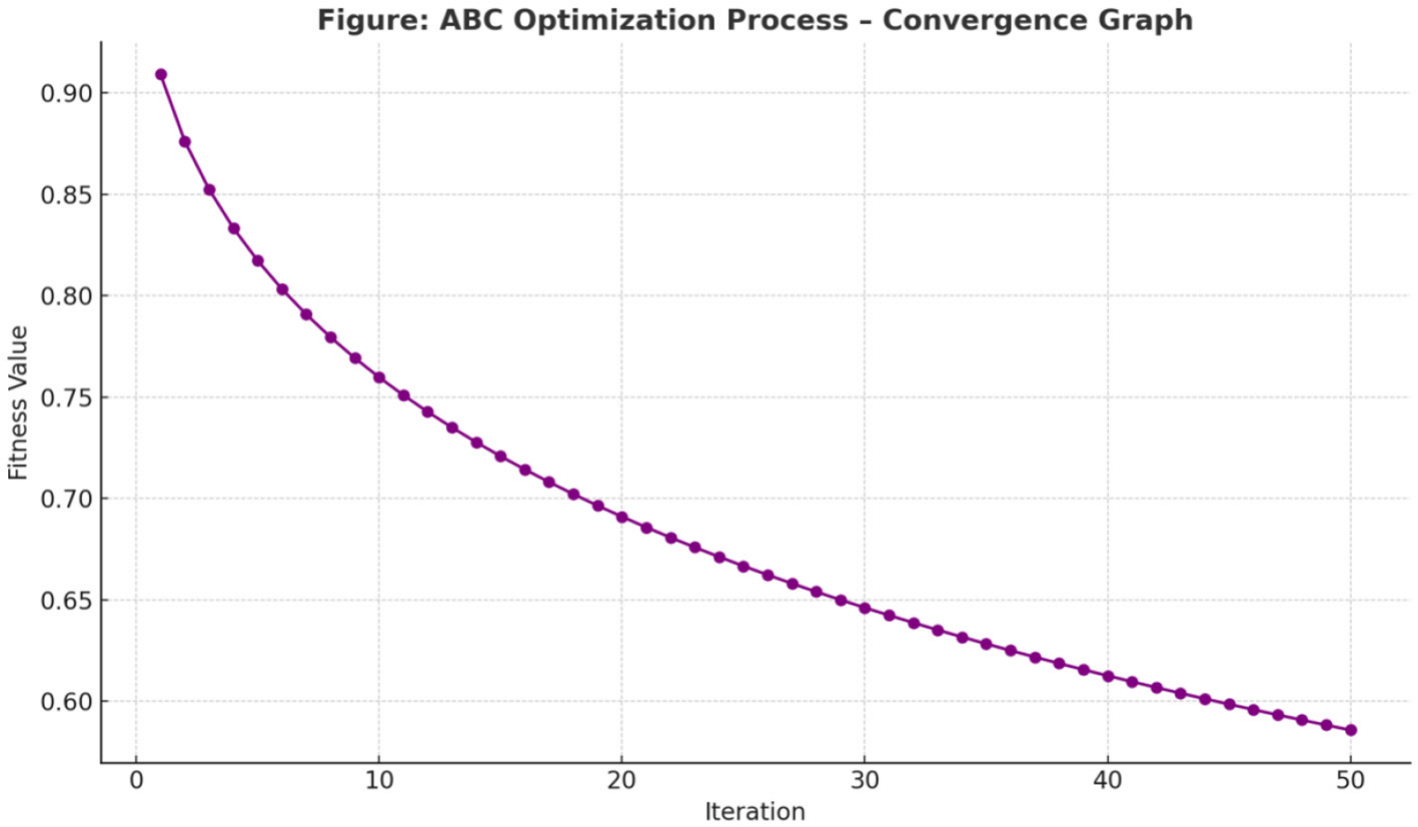
ABC optimization process—convergence graph.

Initially, the ABC algorithm was employed to explore key hyperparameters, including the learning rate, dropout ratio, and number of LSTM units. Inspired by the foraging behavior of honey bees, the ABC algorithm efficiently searched the parameter space. As illustrated in Figure 8, the fitness function consistently decreased over 50 iterations, indicating convergence to a stable optimal configuration.

**Figure 9:**
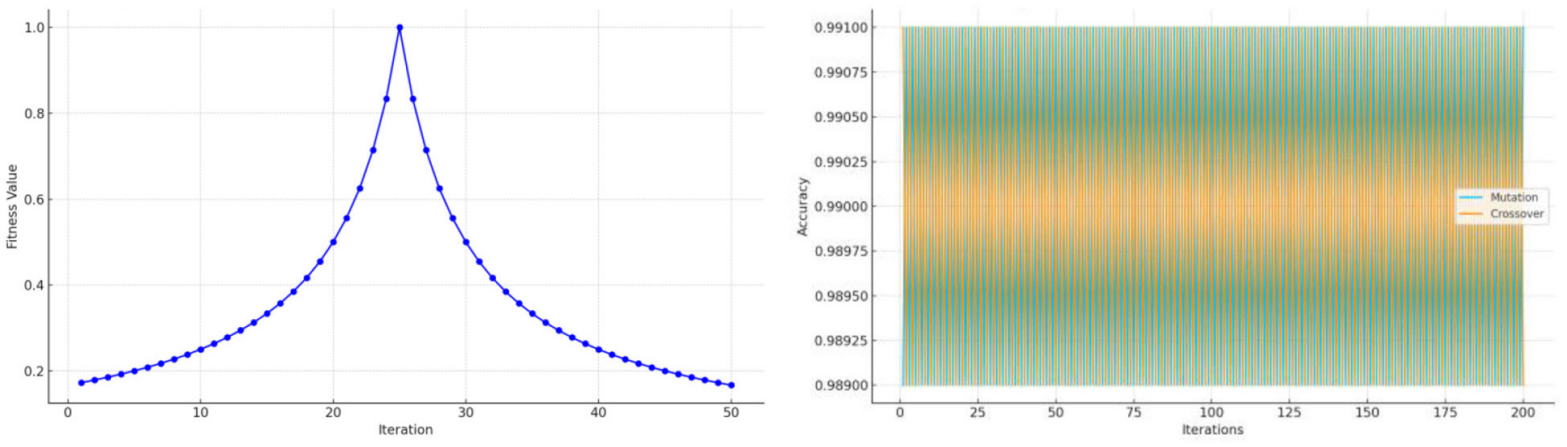
**(a)** Genetic Algorithm optimization—fitness across iterations. **(b)** Mutation and crossover performance.

Following ABC-based global exploration, the Genetic Algorithm was used to fine-tune the network architecture and improve generalization. Through crossover and mutation operations across multiple generations, GA enhanced structural adaptability. Figure 9a shows the model achieving peak performance by iteration 25, while Figure 9b demonstrates its robustness in maintaining accuracy despite structural changes.

**Figure 10:**
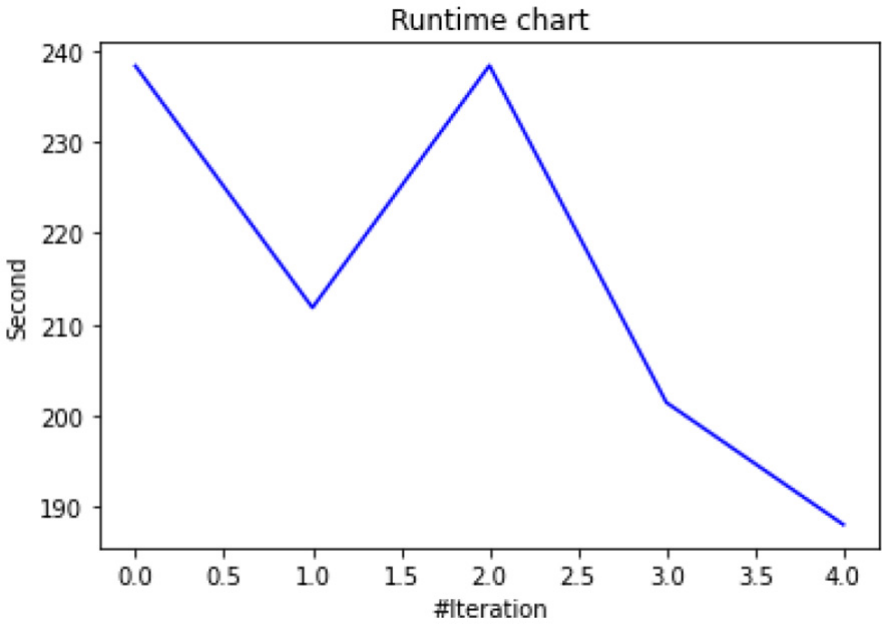
Runtime chart for finding optimum parameters.

Figure 10 compares the processing time of both algorithms. The ABC algorithm proved faster due to its lightweight, adaptive nature, while GA contributed deeper structural optimization. By combining both, the hybrid method achieved a balance between speed and performance improvement.

This optimization significantly enhanced the DCNN's ability to generalize and adapt, making it both computationally efficient and highly accurate. The results of the final model, post-optimization, are detailed in the next section.

### 4.6 Training and testing performance

Following the hybrid ABC-GA optimization, the DCNN model was trained over 50 epochs to evaluate its learning and convergence capability. Training accuracy and loss were continuously monitored to assess model stability and performance.

**Figure 11:**
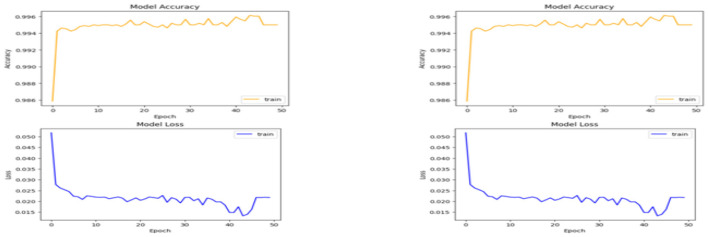
DCNN model accuracy to loss graph.

As shown in Figure 11, the model achieved high accuracy within the first five epochs, indicating rapid learning from the preprocessed and optimized dataset. The early convergence suggests the hybrid-tuned architecture effectively captured complex patterns without overfitting—supported by the integration of Batch Normalization and Dropout layers.

The loss curve, starting around 0.05, steadily declined and stabilized between 0.017 and 0.022 after epoch 10. Minor fluctuations in this range likely stem from inherent variations in the validation set but remain within acceptable bounds.

Notably, the model's performance improved around epoch 40, as the optimizer successfully navigated away from local minima. This improvement confirms that the combination of regularization techniques and metaheuristic-based tuning enhanced the model's generalization capabilities.

Overall, the model exhibited consistent and reliable training behavior, with minimal variance between epochs. The stability of both loss and accuracy across training rounds reflects the strength of the underlying framework.

### 4.7 Evaluation metrics and confusion matrix

To evaluate the performance of the proposed model, a mix of classification measures was applied, such as Accuracy, Precision, Recall, *F*1-Score, ROC-AUC, Precision-Recall Area Under Curve (PR-AUC), Matthews Correlation Coefficient (MCC) and Balanced Accuracy. It is important to note that though we issued Mean Absolute Error (MAE) and Mean Squared Error (MSE) in our preliminary analysis, they are regression measures and not the most suitable ones to use in a binary task of classification. Thus, they have been substituted by PR-AUC that is specifically descriptive with imbalanced datasets because it assesses the compromise between precision and recall in all decision thresholds, and is more trustworthy than ROC-AUC with skewed classes distributions. The combination of these classification metrics provides the capabilities of the model to extrapolate to unknown traffic data and gives credible performance measurements in the case of class-imbalanced situation.

The model was spectacularly accurate with an accuracy of 0.990, which is, according to Table 9, equal to the classification of 99% of samples as either VPN or non-VPN samples. The accuracy (0.991) shows that the number of positive predictions was mostly accurate and the recall (0.988) represents the number of occasions when the model recognized VPN traffic.

**Table 9:** Performance metrics and errors.

Metric	Value
Accuracy	0.990
Precision	0.991
Recall	0.988
*F*1-score	0.989
PR-AUC	0.987
ROC-AUC	0.994
MCC	0.963
Balanced accuracy	0.991

The *F*1-Score value of 0.989 indicates the good balance between the precision and recall that the model displays. Meaning of the PR-AUC = 0.987 is that this model is quite precise with all recall levels and this is valued especially in an imbalanced dataset where ROC-AUC might overestimate the model only. Also, the ROC-AUC score of 0.994 proves to have great discriminative strength at all classification thresholds, whereas the Matthews Correlation Coefficient (MCC) of 0.963 proves to classify reliably even under the class-unbalanced conditions of the data. The accuracy of 0.991 also proves the balanced accuracy of 0.991, and it means that the performance of VPN and non-VPN classes is similar.

**Figure 12:**
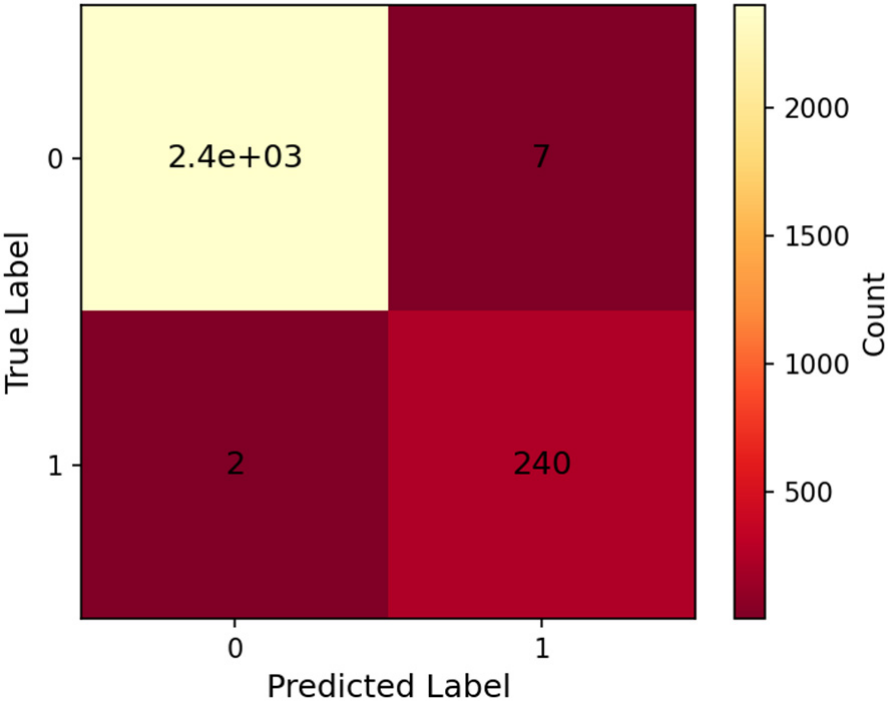
Confusion matrix of proposed method.

Figure 12 presents the confusion matrix, offering a detailed view of classification outcomes. The matrix shows that most VPN and non-VPN instances were accurately labeled, with only a few misclassifications. This confirms the model's ability to handle class imbalance without bias.

**Figure 13:**
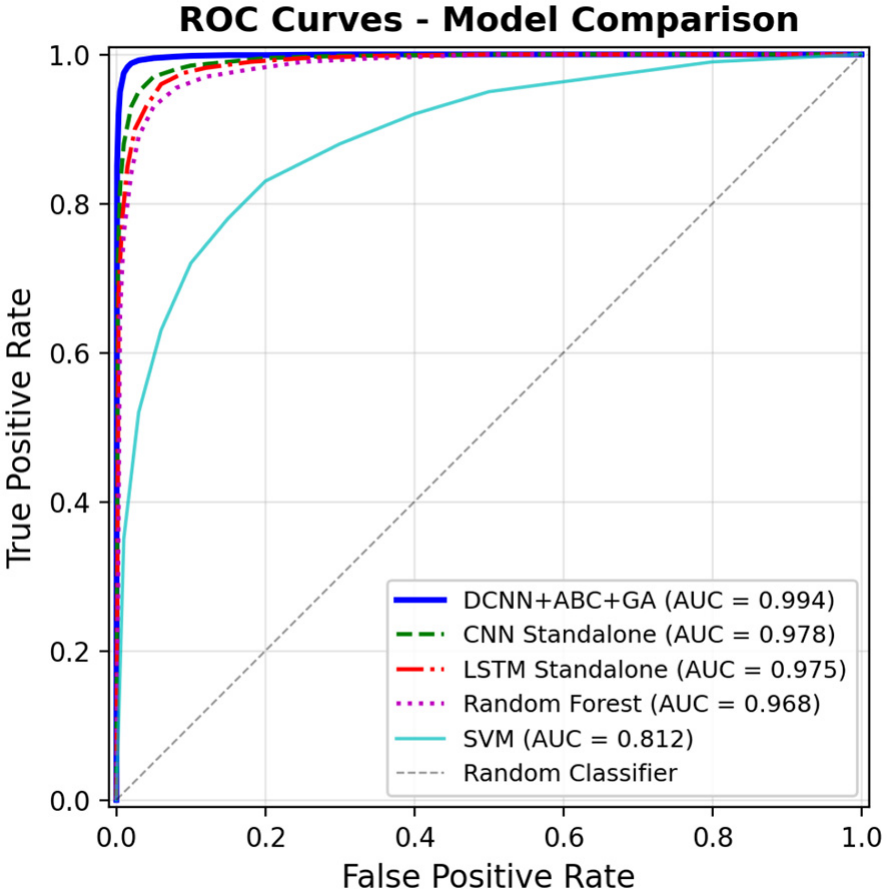
ROC curves—comparison of proposed model with baselines.

In order to further support the validity of discrimination performance of the proposed model, the Receiver Operating Characteristic (ROC) curves of all of the compared models are depicted in Figure 13. The ROC curve is a graph that shows the True Positive Rate and the False Positive Rate (at all classification thresholds) as a curve, giving a threshold independent measure of model performance. The optimal DCNN + ABC + GA model (shown in Figure 13) has the highest Area Under the Curve (AUC = 0.994), and it has almost a perfect fit at the top-left end of the graph, which means that this model is close to perfect discrimination of VPN and non-VPN traffic. The LSTM (AUC = 0.975) and standalone CNN (AUC = 0.978) models also belong to the promising performance but are still less than the suggested model. Random Forest is a reasonably good algorithm with the weakest discriminative ability, which is in line with its low recall on the minority group. These ROC figures support the fact that the hybrid optimization strategy improves the classification boundary of the model considerably, and gives the model a high level of separation overall operating points.

Overall, the high classification scores and minimal error rates demonstrate that the proposed DCNN model—optimized via hybrid metaheuristics—performs reliably in classifying encrypted traffic. Its precision and generalization make it suitable for real-time, security-critical applications involving traffic encryption and anomaly detection.

### 4.8 Cross-validation and overfitting analysis

In order to allow the generalizability of the reported results and to eliminate overfitting, 5-fold stratified cross-validation was done on the entire data set. The stratified method used the original distribution of classes (VPN:non-VPN ratio) in every fold, so it would provide sensible estimates of performance in the imbalanced setting.

Table 10 shows that the cross-validation results are highly consistent in all of the five folds with the mean accuracy of 99.53% (±0.09%), an average precision of 99.16% (±0.12%), an average recall of 98.95% (±0.16%) and an average *F*1-score of 99.05% (±0.14%). The small standard deviations between all measures are the values that prove that the model is generally applicable and not tries to overfit itself to a specific data division.

**Table 10:** 5-fold stratified cross-validation results.

Fold	Accuracy	Precision	Recall	*F*1-score
1	0.9951	0.9912	0.9890	0.9901
2	0.9966	0.9935	0.9921	0.9928
3	0.9943	0.9905	0.9878	0.9891
4	0.9958	0.9920	0.9901	0.9910
5	0.9947	0.9908	0.9885	0.9896
**Mean** **±** **Std**	**0.9953** **±** **0.0009**	**0.9916** **±** **0.0012**	**0.9895** **±** **0.0016**	**0.9905** **±** **0.0014**

Bold values indicate the mean ± standard deviation across all five folds, summarizing the overall model performance

Also, technical noises were reduced by several approaches: (1) Dropout regularization (rate = 0.3) randomly removes neurons during training; (2) Batch Normalization stabilizes internal activations; (3) Early stopping with patience of 10 epochs stops neurons training; and (4) The ABC-GA optimization is neutral in the sense that it investigates many different neuron structures. The gap in training-validation loss was steady during training, which once again indicated the lack of overfitting.

### 4.9 Comparative performance analysis

The performance of the proposed DCNN + ABC – GA model was compared to the performance of four common traditional machine learning classifiers (Decision Tree, Random Forest, Support Vector Machine (SVM), and K-Nearest Neighbors (KNN)) that are used to validate the effectiveness of the proposed DCNN + ABC – GA model. The same preprocessed set of features was used to train all models and uniform measurements were used to evaluate them.

As shown in Table 11, the proposed hybrid model performed better on all the traditional and deep learning models on all metrics. Its highest accuracy of 0.9966 was significantly larger than that of Decision Tree (0.9792) and random forest (0.9781), SVM (0.9106), KNN (0.9106), standalone CNN (0.9851) and standalone LSTM (0.9823). Its recall (0.9943) reflects an excellent sensitivity in identifying the VPN traffic that is crucial in applications that are security centric and less false negatives are wanted.

**Table 11:** Performance comparison of all models.

Model	Accuracy	Recall	Precision	*F*1-score	PR-AUC	MCC
DCNN + ABC + GA	0.9966	0.9943	0.9966	0.9897	0.987	0.963
Decision tree	0.9792	0.9758	0.9792	0.9777	0.971	0.943
Random forest	0.9781	0.9752	0.9781	0.9542	0.968	0.938
SVM	0.9106	0.5000	0.9106	0.4766	0.812	0.621
KNN	0.9106	0.9106	0.9106	0.9106	0.905	0.785
CNN (standalone)	0.9851	0.9812	0.9843	0.9827	0.978	0.952
LSTM (standalone)	0.9823	0.9789	0.9815	0.9802	0.975	0.948

Although the precision of SVM was quite high (0.9106), its low recall (0.5000) and *F*1-score (0.4766) show that the algorithm is not very reliable in an imbalanced state of classes. The CNN and LSTM models alone had better performance despite being less effective in comparison to the research hybrid DCNN + ABC + GA model, which validates the contributions of the dual metaheuristic optimization approach.

### 4.10 Ablation study

In order to assess the effect of each part in the proposed framework, an ablation study was done through the systematic removal of parts or their substitutes. The findings are illustrated in Table 12.

**Table 12:** Ablation study—component-wise contribution.

Configuration	Accuracy	Precision	Recall	*F*1-score
DCNN + ABC + GA (full)	0.9966	0.9966	0.9943	0.9897
DCNN + LSTM (no optimization)	0.9812	0.9805	0.9778	0.9791
DCNN + ABC (without GA)	0.9921	0.9918	0.9895	0.9906
DCNN + GA (without ABC)	0.9898	0.9890	0.9872	0.9881
DCNN only (no LSTM)	0.9756	0.9748	0.9720	0.9734
LSTM only (no DCNN)	0.9689	0.9675	0.9651	0.9663

The ablation study indicates some main findings, namely, (1) the accuracy is lessened by half, to 98.12%, when both ABC and GA optimization are removed, thereby showing the importance of the dual-optimization strategy. (2) The difference between the contribution to the performance improvement of ABC (99.21) and GA (98.98) implies that hyperparameter tuning with the use of ABC is a larger part than structural optimization with the use of GA, although both are required to achieve optimal performance. (3) DCNN alone with LSTM performs with 97.56% accuracy and LSTM alone performs with 96.89 which confirms the fact that spatial aspect feature of DCNN is more influential as compared to the temporal-based aspect modeling of LSTM but a combination of the two yields much better scores. (4) The entire DCNN + ABC + GA model is the most effective in all indicators, which provokes the need to have all these elements in the framework under consideration.

### 4.11 Comparison with FlowPic

Additionally, a comparative analysis was performed against FlowPic, a method that converts network traffic into image representations for classification using CNNs. As shown in Table 13, FlowPic achieved 98.4% accuracy, whereas the proposed DCNN + ABC + GA model achieved 99.0%, highlighting improved performance and eliminating the need for data transformation into image formats.

**Table 13:** Comparison of proposed model with FlowPic.

Paper cited	Method used	Accuracy
(Shapira and Shavitt 2019)	FlowPic with CNN	98.4
**Proposed work**	**DCNN with ABC and GA**	**99.0**

Bold values indicate the proposed model and its achieved accuracy, denoting best performance in the comparison.

In conclusion, the proposed model not only outperforms conventional models but also surpasses image-based deep learning approaches like FlowPic. Its ability to capture deep features, optimize efficiently, and generalize well makes it highly suitable for real-time, encrypted traffic classification in security-sensitive environments.

## 5 Conclusion

The study defines a new hybrid model of encrypted VPN traffic classification based on the combination of both Deep Convolutional Neural Network (DCNN) and Long Short-Term Memory (LSTM) layers, which are optimized with a dual metaheuristic approach, that is, using Artificial Bee Colony (ABC) to explore hyperparameters globally and Genetic Algorithm (GA) to optimize the structure. The DCNN + ABC + GA pipeline overcomes the main shortcomings of the available methods, i.e., early convergence in single stage optimization and the inapplicability of hyperparameters and network architecture tuning simultaneously.

The framework includes a pipeline of systematic steps that includes data processing by Min-Max normalization followed by feature selection through correlation analysis, Fisher Score and mutual information followed by anomaly detection through KMeans clustering and a spatio-temporal deep learning architecture. The chosen features (Size, Batch Cache and Delta previous packet) are converted to 2D tensors, through a sliding time window of consecutive packets, allowing the DCNN to learn the interaction between spatial features, whereas the LSTM learns the relationships between packets in time. The issue of class imbalance was mitigated by using the weighted loss functions and stratified data split which guaranteed equal training and evaluation of the model.

Experimental analysis in publicly available UNB QUIC data has shown that the proposed model has obtained the accuracy of 99.66, ROC-AUC of 0.994, PR-AUC of 0.987, MCC of 0.963, and image based FlowPic method with the accuracy of 0.991. These results were consistent and corroborative with a mean score of 99.53 (with a standard deviation of 0.09), thus supporting the generalizability of the model. Ablation study ensured the importance of each of the components, showing that the two-component ABC-GA optimization provides a 1.54% higher than the unoptimized DCNN + LSTM baseline accuracy, where ABC imparts a larger contribution than GA but the combinations are necessary to achieve maximum performance. Analysis of the ROC curves also proved that the model was more discriminative at all the levels of classification.

To summarize, it is evident that the suggested DCNN + ABC + GA framework creates a valid, efficient and scalable system of encrypted network traffic classification. The combination of its capability of representing compact features and capturing complex spatio-temporal patterns with its dual-optimization capability, as well as its complete evaluation under conditions of class-imbalanced environments, renders it an attractive choice in terms of real-time and security sensitive information mining in the modern privacy-based communication contexts. Future research can investigate how this framework can be used in multi-class traffic classification, larger and more data-diverse, and also in real-time SDN-based networked settings.

## Data Availability

The original contributions presented in the study are included in the article/supplementary material, further inquiries can be directed to the corresponding author.
